# Src activates Abl to augment Robo1 expression in order to promote tumor cell migration

**DOI:** 10.18632/oncotarget.126

**Published:** 2010-07-20

**Authors:** P. Raaj Khusial, Bhaskar Vadla, Harini Krishnan, Trudy F. Ramlall, Yongquan Shen, Hitoshi Ichikawa, Jian-Guo Geng, Gary S. Goldberg

**Affiliations:** ^1^ Molecular Biology Department, University of Medicine and Dentistry of New Jersey, Stratford, NJ 08084, USA; ^2^ Graduate School of Biomedical Sciences, University of Medicine and Dentistry of New Jersey, Stratford, NJ 08084, USA; ^3^ Department of Biochemistry and Program in Structural Biology, Weill Medical College of Cornell University, New York, New York 10065, USA; ^4^ Genetcis Division, National Cancer Center Research Institute, 5-1-1 Tsukiji, Chuo-ku, Tokyo 104-0045, Japan; ^5^ Vascular Biology Center, Division of Hematology, Oncology, and Transplantation, Department of Medicine, University of Minnesota Medical School, Minneapolis, Minnesota 55455, USA

**Keywords:** Tyrosine kinase, Src kinase Abl kinase, Robo1, Rho GTPase, Cancer, Cell migration, Cell communication, Connexin

## Abstract

Cell migration is an essential step in cancer invasion and metastasis. A number of orchestrated cellular events involving tyrosine kinases and signaling receptors enable cancer cells to dislodge from primary tumors and colonize elsewhere in the body. For example, activation of the Src and Abl kinases can mediate events that promote tumor cell migration. Also, activation of the Robo1 receptor can induce tumor cell migration. However, while the importance of Src, Abl, and Robo1 in cell migration have been demonstrated, molecular mechanisms by which they collectively influence cell migration have not been clearly elucidated. In addition, little is known about mechanisms that control Robo1 expression. We report here that Src activates Abl to stabilize Robo1 in order to promote cell migration. Inhibition of Abl kinase activity by siRNA or kinase blockers decreased Robo1 protein levels and suppressed the migration of transformed cells. We also provide evidence that Robo1 utilizes Cdc42 and Rac1 GTPases to induce cell migration. In addition, inhibition of Robo1 signaling can suppress transformed cell migration in the face of robust Src and Abl kinase activity. Therefore, inhibitors of Src, Abl, Robo1 and small GTPases may target a coordinated pathway required for tumor cell migration.

## INTRODUCTION

Tumor cell migration leads to metastasis, which is responsible for about 90% of deaths caused by cancer [1]. Signaling by a number of different growth factors, including EGF and PDGF, can activate the Src and Abl kinases in order to promote tumor cell invasion and metastasis [2,3]. Src and Abl are nonreceptor tyrosine kinases, that can inturn phosphorylate a variety of substrates, including Cas, Crk, and paxillin, to initiate cell spreading and migration [4-6]. Src phosphorylates Abl [2], which associates with actin and the Rho family of GTPases to modify effectors including N-WASP to promote cytoskeletal reorganization and cell motility [3,7,8]. However, Src also relies on parallel pathways to induce tumor cell growth and migration [9,10].

The Abl kinase can phosphorylate the Robo1 receptor [11]. Robo1 is a transmembrane receptor of the immunoglobulin family [7,12]. Upon binding to its ligand, Slit2, Robo1 works with the Abl kinase to rearrange the actin cytoskeleton and induce cell migration [13,14]. Indeed, cell migration can be inhibited by blocking Robo1 receptor activity with a monoclonal antibody to the extracellular domain of the protein [14]. Hence, the Abl kinase provides a link between Robo1 and the actin cytoskeleton. However, the role of Src kinase activity in this pathway has not been defined.

Here, we describe a molecular relationship between Src, Abl, and Robo1 that promotes tumor cell migration. Our data indicate that Src activates the Abl kinase, which in turn, stabilizes the expression of Robo1 and increases GTPase activity to promote tumor cell migration. These results demonstrate how monoclonal antibodies and kinase inhibitors may be used to target specific components of the Src/Abl/Robo1 pathway to prevent tumor cell migration at multiple steps.

## RESULTS

### Src augments Robo1 production in transformed cells

**Fig. 1:**
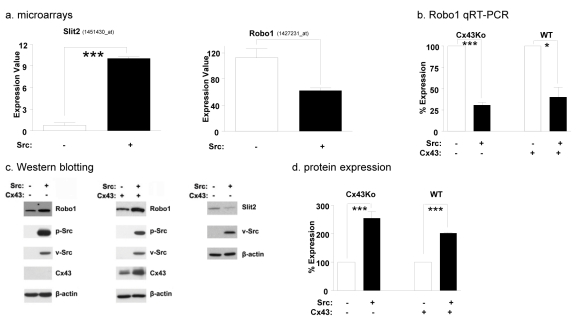
Src reduces Robo1 mRNA levels, but increases Robo1 protein levels. **(a)** mRNA was extracted from nontransformed and Src transformed Cx43Ko cells and examined by microarray analysis. Expression values of Affymetrix probe sets representing Slit2 and Robo1 are shown (mean+SEM, n=2). **(b)** Robo1 mRNA expression was examined by qRT-PCR analysis of RNA purified from nontransformed and Src transformed cells obtained from wild type embryos (WT) or homozygous null Cx43 knockout embryos (Cx43Ko) as indicated. Data are shown as the percent of nontransformed cells (mean+SEM, n=2). **(c)** Western blotting was used to compare the expression of Robo1, total Src, active Src, Cx43, Slit2, and β-actin in nontransformed and Src transformed cells. Robo1 protein expression was quantitated from Western blot analysis of nontransformed and Src transformed cells and shown as percent of nontransformed cells (mean+SEM, n=3). Single and triple asterisk indicate p values less than 0.5 and 0.005, respectively (by t-test).

The Src kinase activates a variety of pathways that promote cell migration [9,10]. For example, data from previous experiments indicate that Src induces the expression of Slit2 [15] which binds to its receptor, Robo1, to promote cell migration [13,14,19,22]. We utilized expression microarrays to further investigate the effect of Src kinase activity on Slit2 mRNA expression. As shown in Figure 1a, these data indicate that Src transformed cells expressed over 4 times more Slit2 mRNA than nontransformed cells.

As stated above, Slit2 is a secreted protein that binds to the Robo1 receptor [13,14,19,22]. Upon Slit2 binding to Robo1 the activity of the Rho GTPases are regulated to rearrange the actin cytoskeleton and induce cell motility [7]. Therefore, if Slit2 were promoting tumor cell migration, Src may also be expected to increase Robo1 expression in transformed cells. However, Affymetrix microarray data indicate that Src transformed cells contained less Robo1 mRNA than nontransformed cells (Figure 1a). The suppression of Robo1 mRNA expression by Src was also confirmed by qRT-PCR as shown in Figure 1b.

Src phosphorylates Cx43 to block gap junctional communication between transformed cells [23,24]. Indeed, Cx43 can act as a tumor suppressor in a number of cell types including mammary carcinoma [25,26] and glioma cells [27-29]. In contrast, Robo1 activity can promote glioma cell migration [30] and metastasis of breast cancer cells to the brain [31]. Thus, we sought to analyze the role of Robo1 in the migration of wild type mouse embryonic fibroblasts that express Cx43, as well as brain cells from Cx43 knockout mice (Cx43Ko cells). Cx43 did not affect the ability of Src to decrease Robo1 expression at the mRNA level. As shown in Figure 1b, results from qRT-PCR revealed that both Src transformed Cx43Ko and wild type cells expressed less than half as much Robo1 mRNA as nontransformed cells. However, in contrast to mRNA expression, Src appeared to augment Robo1 protein expression in both Cx43Ko and wild type cells by about 2 fold (Figure 1c-1d).

**Fig. 2:**
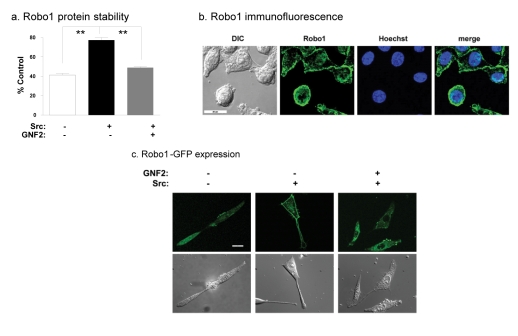
Src stabilizes Robo1 protein which localizes to the plasma membrane. **(a)** Protein from cyclohexamide treated nontransformed cells, Src transformed cells, and Src transformed cells treated with the Abl kinase blocker GNF-2 were analyzed by Western blotting to evaluate the effects of Src and Abl on Robo1 protein stability. Data are shown as the percent of Robo1 protein after 24 hours of cyclohexamide treatment compared to untreated controls (mean+SEM, n=2). **(b)** Immunofluorescence microscopy was used to visualize Robo1 (green) and nuclei (blue) in Src transformed cells. DIC images and merged images are also shown as indicated (bar = 20 microns). (c) GFP tagged Robo1 was visualized by fluorescence microscopy in nontransformed cells, Src transformed cells, and Src transformed cells treated with GNF-2. DIC images are also shown as indicated (bar = 20 microns). Double asterisks indicate p values less than 0.01 compared to controls (by t-test).

Taken together, qRT-PCR and protein analyses indicate that Src decreased Robo1 mRNA levels while increasing Robo1 protein levels (Figure 1a-1c). These data suggest that Src can stabilize Robo1 protein in transformed cells. To investigate this, we measured the effects of the protein synthesis inhibitor cyclohexamide on the stability of Robo1 in nontransformed and Src transformed cells. After 24 hours of cyclohexamide treatment, about 75% of Robo1 remained in Src transformed cells compared to about 40% in nontransformed cells (Figure 2a). Thus, Src appeared to increase Robo1 protein stability in transformed cells by about 2 fold.

As shown in Figure 2b, Robo1 was found at the membrane of Src transformed cells where it may act as a functional receptor to promote tumor cell migration and invasion. In addition, a Robo1-GFP fusion construct was also utilized to examine the location of Robo1 in nontransformed and Src transformed cells. As seen in Figure 2c, this Robo1-GFP protein was found mostly at the plasma membrane of Src transformed cells. In contrast, Robo1-GFP was found more diffusely through the cytoplasm in nontransformed cells and transformed cells treated with an Abl kinase blocker (GNF-2), as discussed below.

### Src utilizes Robo1 to promote cell migration

**Fig. 3:**
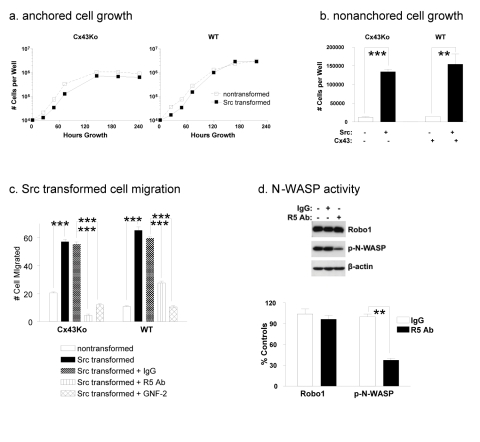
Src utilizes Robo1 to promote cell migration. Cells obtained from wild type (WT) or homozygous null Cx43 knockout (Cx43Ko) mouse embryos were transfected with v-Src or the empty parental vector and plated (10,000 per well) were plated on standard or ultra low attachment culture dishes to evaluate **(a)** anchored and **(b)** nonanchored cell growth. Cell numbers were determined by Coulter counter at the indicated time points for anchored cells or at 7 days for nonanchored cells. Data are shown as number of cells per well at the indicated time points (mean+SEM, n=3). **(c)** Cell migration was examined by a wound healing assay on nontransformed cells and Src transformed cells treated with IgG control antiserum, R5 antiserum to block Robo1 activity, or GNF-2 Abl kinase blocker. Migration was quantitated as the number of cells that entered a 1.8 mm2 area of the wound during 24 hours (mean+SEM, n=5). **(d)** Src transformed cells were treated with R5 antibody or control antiserum and analyzed by Western blotting for Robo1, active N-WASP (p-N-WASP), or β-actin. N-WASP activity was then quantitated and shown as percent of untreated control cells (mean+SEM, n=2). Experiments were performed with Cx43Ko and WT cells, with results from Cx43Ko cells shown in panel d. Double and triple asterisk indicate p values less than 0.01 and 0.005 compared to controls, respectively (by t-test).

Src transformed Cx43Ko and wild type cells achieved similar levels of anchorage independence (see Figure 3a and 3b). In addition, Src significantly increased the motility of both Cx43Ko and wild type cells. Src transformed Cx43Ko and wild type cells migrated about 3 fold and 6 fold more than nontransformed cells, respectively (Figure 3c and Supplementary Figure S1).

As mentioned above, Slit2/Robo1 signaling can promote glioma cell migration [30] and metastasis of breast cancer cells to the brain [31]. We utilized a neutralizing antibody (R5 Ab) to examine the effects of Robo1 on the motility of Src transformed cells. This monoclonal antibody targets the first immunoglobulin domain of Robo1 to block activation of Robo1 signaling [14,19]. As described above, Src significantly increased the migration of Cx43Ko and wild type cells. As shown in Figure 3c, application of R5 Robo1 antiserum suppressed the migration of these Src transformed cells by at least 2 fold. As shown in Figure 1c, in contrast to mRNA levels, Src transformed cells actually expressed less Slit2 protein than nontransformed cells. In addition, conditioned medium from Src transformed cells did not affect the migration of nontransformed cells, indicating that Slit2 induction was not sufficient to account for the increased migration of Src transformed cells (see Supplementary Figure S2). Thus, these data indicate that Src augmented Robo1, but not Slit2, production to promote tumor cell migration. However, the data also clearly indicate that Robo1 induced migration in a Slit2 dependent manner.

### Src activates Abl to augment Robo1 expression and GTPase activity

Many axon guidance receptors, including Robo1, regulate the Rho family of GTPases to affect cell motility. N-WASP is an effector through which Rho GTPases regulate the actin cytoskeleton [7]. Effects of R5 antiserum are also evident on N-WASP activity. Application of the R5 antiserum reduced N-WASP activity by over 60% (Figure 3d).

**Fig. 4:**
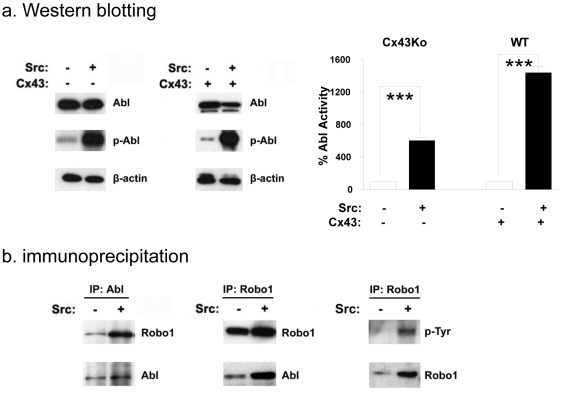
Src associates and activates Abl in transformed cells. **(a)** Western blotting was used to compare the expression of Abl, active Abl, and β-actin in nontransformed and Src transformed Cx43Ko or wild type (WT) cells. Abl kinase activity was quantitated and shown as percent of nontransformed cells (mean+SEM, n=2). **(b)** Protein from nontransformed and Src transformed cells was immunoprecipitated with either anti-Abl or anti-Robo1 antibody, and analyzed by Western blotting with antiserum specific for Robo1, Abl, or phosphotyrosine as indicated. Triple asterisks indicate p values less than 0.005 compared to controls (by t-test).

Robo1 interacts with the Abl kinase to initiate cytoskeleton rearrangement [11,32]. However, in contrast to this proposed role of Abl as a Robo1 effector, we hypothesized that Src activates Abl to stabilize Robo1 expression in transformed cells. As seen in Figure 4a, although Abl protein expression levels were similar in both Src transformed and nontransformed cells, Src increased the activity of Abl in transformed cells by several fold.

Results from immunoprecipitation studies indicate that Robo1 and Abl associate with each other and that Robo1 is phosphorylated on tyrosine in Src transformed cells. Lysates from Src transformed and nontransformed cells were immunoprecipitated with anti-Robo1 antiserum and immunoblotted with anti- Abl antibody. Reciprocal experiments were also performed; lysates were immunoprecipitated with anti-Abl antibody and immunoblotted with anti-Robo1. As shown in Figure 4b, results from these experiments indicate that Robo1 and Abl associated with each other in both Src transformed and nontransformed cells. However, as shown in Figure 4b, phosphotyrosine residues were detected in Robo1 from transformed cells, but not nontransformed cells. These data suggest that Src or Abl can phosphorylate Robo1 in transformed cells.

**Fig. 5:**
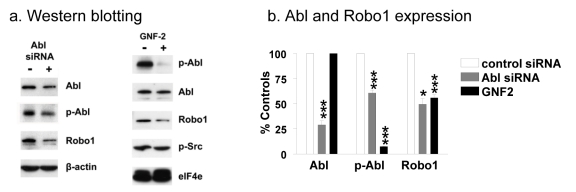
Src activates Abl to augment Robo1 protein levels. **(a)** Src transformed Cx43Ko cells were transfected with siRNA against Abl, or treated with the Abl kinase blocker GNF-2, and analyzed by Western blotting for Abl, active-Abl, Robo1, active Src, elF4e, and β-actin as indicated. **(b)** Abl expression, Abl activity, and Robo1 expression were then quantitated and shown as percent of control cells (mean+SEM, n=2). Single and triple asterisks indicate p values less than 0.5 and 0.005, respectively (by t-test).

We utilized siRNA and a chemical kinase blocker to determine if Src utilized Abl to augment Robo1 protein expression. As shown in Figure 5, Abl siRNA effectively silenced Abl protein expression and kinase activity in Src transformed cells by about 70% and 40%, respectively. This resulted in a similar decrease in Robo1 expression. Moreover, treatment of Src transformed cells with the Abl kinase blocker GNF-2 did not affect Abl protein levels, but reduced Abl kinase activity by over 90% and, consequently reduced Robo1 expression by about 50% (Figure 5).

Cyclohexamide treatment shown in Figure 2a also indicates that Src utilized Abl to stabilize Robo1 at the protein level. After 24 hours of cyclohexamide treatment, about 75% of Robo1 remained in Src transformed cells compared to about 40% in nontransformed cells or transformed cells treated with the Abl blocker GNF-2 (Figure 2a). As shown in Figure 2c, inhibition of Abl kinase activity by GNF-2 also resulted in intracellular cytoplasmic Robo1 localization similar to that seen in nontransformed cells, as opposed to plasma membrane localization seen in Src transformed controls. These data suggest that Abl may stabilize Robo1 at the plasma membrane. It should be noted that GNF-2 is highly specific for the Abl kinase [33], having no detectable effect on Src kinase activity at the concentrations used here (see Figure 5a). Thus, Robo1 expression at the cell membrane appeared to depend on Abl kinase activity in Src transformed cells.

**Fig. 6:**
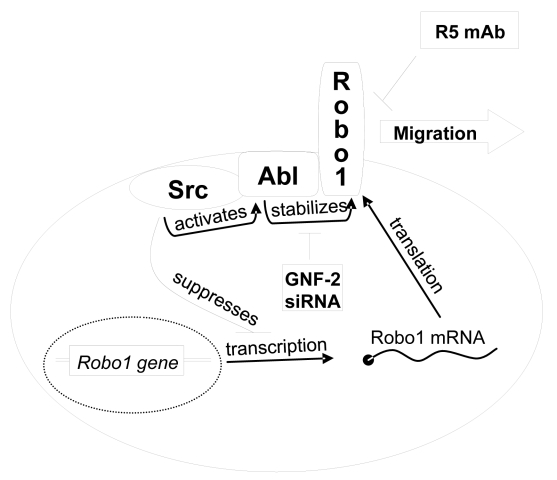
Schematic diagram illustrating how Src activates Abl to stabilize Robo1 expression in order to promote cell migration. This pathway can be suppressed by reagents that target Abl production (siRNA), Abl activity (GNF-2), or Robo1 activation (R5 Ab).

The effects of abrogating Abl kinase activity on cell migration were also examined. As shown in Figure 3c and Supplementary Figure S1, these data indicate that Abl kinase activity promoted the migration of Src transformed cells. Src transformed cells treated with GNF-2 migrated about 5 fold less than control cells. Taken together, these data indicate that Src activated Abl to induce Robo1 expression in order to increase migration of transformed cells as illustrated in Figure 6.

Abl kinase activity has been implicated in the progression of non small cell lung cancer [34].

**Fig. 7:**
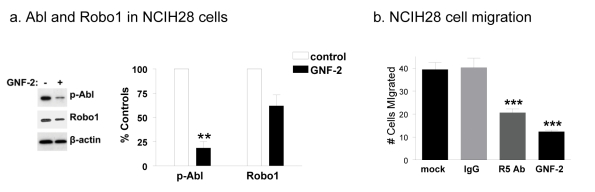
Src activates Abl to promote Robo1 expression and migration of human mesothelioma cells. **(a)** Western blotting was used to compare the expression of active Abl, Robo1, and β-actin in NCIH28 mesothelioma cells treated with R5 antiserum to block Robo1 activity or GNF-2 Abl kinase blocker. Abl kinase activity and Robo1 levels were quantitated and shown as percent of nontransformed cells (mean+SEM, n=2). **(b)** Cell migration was examined by a wound healing assay on NCIH28 cells treated with GNF-2 or R5 antibody, as well as controls, as indicated. Migration was quantitated as the number of cells that entered a 1.8 mm^2^ area of the wound during 24 hours (mean+SEM, n=5). Double and triple asterisk indicate p values less than 0.01 and 0.005, respectively (by t-test).

As shown in Figure 7a, NCI-H28 mesothelioma cells exhibited robust Abl activity and Robo1 protein expression which were reduced by over 80% and 40%, respectively, by treatment with GNF-2. As shown in Figure 7b, GNF-2 and R5 antiserum reduced NCI-H28 cell migration by about 50% and 75%, respectively (also see Supplementary Figure S3). These studies indicate that the Abl kinase can augment Robo1 protein levels to promote the migration of mesothelioma cells.

**Fig. 8:**
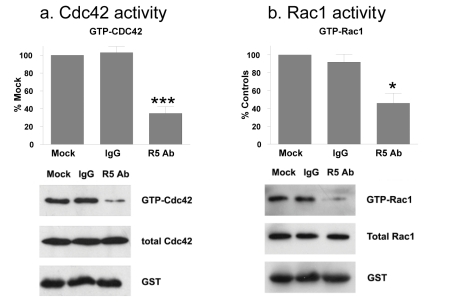
Robo1 signaling activates Rho GTPases in transformed cells. Src transformed cells were treated with R5 antibody or control antiserum (IgG) and examined for total and activated Cdc42 and Rac1 GTPases in **panels a and b**, respectively. Western blotting was performed to detect active (GTP bound) Cdc42 and Rac1, total Cdc42 and Rac1, and GST. Levels of active Cdc42 and Rac1 were quantitated and shown as the percent of untreated control cells (mean+SEM, n=3). Experiments were performed with Cx43Ko and wild type cells, with results from Cx43Ko cells shown. Single and triple asterisks indicate p values less than 0.5 and 0.005, respectively (by t-test).

Robo1 modulates Rho GTPase activity to affect the actin cytoskeleton. In particular, Cdc42 and Rac1 associate with N-WASP to regulate actin remodeling leading to filopodia and lamellipodia movement required for cell migration [7,31]. As shown in Figure 3d, Robo1 appeared to activate N-WASP in Src transformed cells. We therefore, sought to determine if Cdc42 or Rac1 activity were affected during Robo1 mediated migration of Src transformed cells. As shown in Figure 8, while total Cdc42 and Rac1 protein levels were not affected, R5 antiserum reduced levels of GTP bound Cdc42 and Rac1 by approximately 50%. These data are consistent with a role for Cdc42 and Rac1 in tumor cell migration mediated by Robo1.

## DISCUSSION

Src and Abl are non-receptor tyrosine kinases that play critical roles in tumor cell migration leading to invasion and metastasis [5,34]. Activities of these kinases have been associated with many types of human cancers, including tumors of the colon, breast, pancreas, lung, blood, and brain [34,35]. Indeed, Src and Abl kinase inhibitors may serve as chemotherapeutic medicines [36-39].

Studies have implicated Robo1 in liver, breast, and brain cancers [30,31,40,41]. Moreover, Robo1 signaling can augment tumor angiogenesis [13,22]. Plattner *et al*. have reported that Src activates the Abl kinase to promote cell migration [2]. In addition, studies suggest that the Abl kinase associates with Robo1 [11,32,42]. However, actual mechanisms by which Src, Abl, and Robo1 work together to promote cell migration have not been defined. Here, we provide evidence that Robo1 plays an important role in tumor cell migration induced by Src and Abl.

Many axon guidance receptors including Robo1 regulate the Rho family of GTPases to effect changes in motility. Rho GTPases are members of the Ras superfamiliy. There are over 20 mammalian Rho GTPases [43]. When bound to GTP, these GTPases target effectors to modify the actin cytoskeleton. In particular, 3 Rho GTPases, Rho, Rac, and Cdc42 activate members of the Wiskott-Aldrich syndrome proteins (WASPs) and WASP verprolin homologous proteins (WAVEs) which, in turn, activate actin polymerization factors, including Arp2/3 and formin, to cause elongation and branching of actin fibers [44- 46]. Our data indicate that Slit2 binds to Robo1 in order to activate Cdc42 and Rac1 GTPases, which leads to N-WASP activation.

Previous studies suggest that Abl can inhibit Robo1 signaling [11], and that Robo1 can decrease Cdc42 activation during axon guidance and neuronal migration [7]. However, in contrast to previous observations from studies in developing drosophila and mouse embryos [7,11], results from our studies demonstrate that Src activates the Abl kinase, which in turn stabilizes Robo1 protein to increase Cdc42 and Rac1 GTPases activity to promote transformed cell migration. Indeed, suppression of Abl activity (with siRNA or GNF-2) decreased Robo1 protein levels and inhibited the migration of transformed cells. In addition, inhibition of Robo1 signaling by monoclonal antiserum inhibited tumor cell migration without affecting Abl kinase activity. As illustrated in Figure 6, these studies suggest that monoclonal antibodies and kinase inhibitors may be used to target specific components of the Src/Abl/Robo1 pathway to prevent tumor cell migration at multiple steps.

Our data indicate that the induction of cell migration by Robo1 is highly regulated. Suppression of Robo1 mRNA production by Src, coupled with the need for Abl to stabilize Robo1 protein at the plasma membrane ensure that cell migration results from a response to a combination of appropriate stimuli. Thus, two oncogenic kinases, Src and Abl, must work together to promote Robo1-mediated cell migration.

## MATERIALS AND METHODS

### Cells and treatments

Cells obtained from wild type (WT) or homozygous null Cx43 knockout (Cx43Ko) mouse embryos were transfected with v-Src or the empty parental vector and cultured as described [15-18]. For some experiments, nontransformed and Src transformed Cx43Ko cells were transfected with a Robo1-GFP expression vector, which was generated by cloning the entire coding region of Robo1 (accession #: AF040990) into the Nhe1-Sac1 sites of pEGFP-N1 (Clontech). NCIH28 mesothelioma cells were obtained from the ATCC. For some experiments, cells were treated overnight with 50 μg/ml cyclohexamide (Sigma, C7698), 60 nM R5 antibody to the extracellular region of Robo1, 60 nM control IgG antibody [14,19], or 40 μM GNF-2 Abl kinase inhibitor (Calbiochem, 197221).

### Expression microarrays

Gene expression in nontransformed and Src transformed Cx43Ko cells was examined by microarray analysis with Mouse Genome 430 2.0 Arrays (Affymetrix) as previously described [15,16,18]. These arrays contain approximately 45,000 probe sets which represent over 30,000 genes. Affected probe sets displayed a difference of at least 4 fold between transformed and nontransformed cells, or at least a 2 fold change with p<0.05 by t-test with n=2. All comparisons were done with cells from parallel cultures to control for variability in reagents or experimental conditions. Expression analysis was performed with Vector Xpression software 4.0 (Invitrogen).

### siRNA transfection

Small interference RNA (siRNA) targeted to Abl (Santa Cruz Biotechnology, SC29844) or siCONTROL nontargeting siRNA (Dharmacon D00120613) were transfected into cells at a final concentration of 100 nM with Lipofectamine 2000 (Invitrogen, 12252-011).

### qRT-PCR

Total RNA was extracted from cells using TRI RNA Isolation Reagent (T9424, Sigma). cDNA was then synthesized using Protoscript first strand cDNA synthesis Kit (E6500S, BioLabs). Quantitative RTPCR amplification of Robo1 and Actin cDNA was performed with iQ SYBR Green Supermix (BioRad) using primers for Actin (5'-CCCAGAGCAAGAGAGG-3' and 5'-GTCCAGACGCAGGAT-3') and Robo1 (5'- GAGGTAGCTATACTACGGGATGAC-3' and 5'- CAGATGTAGTAGCCGACATCAGAC-3'). SYBR green emission intensities were measured during the amplification reaction with an iCycler detection system (Applied Biosystems 7800).

### Western blotting and Immunoprecipitation

For co-immunoprecipitation studies, cells were lysed in CSK buffer (100mM NaCl, 1.5mM MgCl_2_, 10mM pipes pH 6.8) containing 0.5% Triton-X-100, 1mM sodium vanadate (Sigma, S6508), 1mM PMSF (Sigma, P7626), 50mM NaF (Sigma, S7920) and 1% Protease inhibitor cocktail for mammalian cells (Sigma, P2417). For phosphotyrosine studies, cells were lysed in 20 mM Tris-HCl (pH 6.9) containing 125 mM NaCl, 1 mM EDTA, 1 mM EGTA, 0.75% Triton X-100, 1 mM β-glycerolphosphate, 50 mM NaF, 1 mM Na_3_VO_4_, 1mM PMSF and 10 μg/ml proteinase inhibitor mixture (Sigma, catalog number P2714). Lysates were clarified by centrifugation and incubated with either Robo1 (Santa Cruz Biotechnology, 16612) or Abl (Santa Cruz Biotechnology, SC 23) at 4°C overnight. Protein A/G (Santa Cruz) or protein G (Pierce, 20398) were added and the reaction was incubated for an additional 2h. Immune complexes were washed with lysis buffer, and eluted by boiling the samples in sample buffer (7.5% SDS, 80mM DTT, 0.25M Tris pH6.8, 8.25% glycerol, 0.01% bromophenol blue). Total protein was obtained from cells lysed in buffer containing 62.5mM Tris-HCl (pH6.8), 50mM DTT, 2% SDS and 10% glycerol. Proteins were resolved by gel electrophoresis, transferred to Immobilion-P membranes (Millipore IPVH00010), and subjected to immunoblotting with antisera specific for Robo1 (Abcam, ab7279), active Src kinase (phosphorylated at Y416) (Cell Signaling Technology, 2101), v-Src (Upstate Biotechnologies, 05-185), Cx43 (BD Transduction Laboratories, 610061), Abl (Cell Signaling Technologies, 2862), active Abl kinase (phosphorylated at Y245) (Cell signaling Technologies, 2861S), active N-WASP (phosphorylated at S484 and S485) (Chemicon International, AB1964), Cdc42 (Abcam, ab41429), Rac1 (BD Transduction Laboratories, 610650), elF4E (Cell Signaling Technologies, 9742), Slit2 (Millipore, O94813), β-actin (Sigma, A1978), phosphotyrosine (Cell Signaling Technology, 9411), or glutathione Stransferase (GST). Signal was detected with appropriate HRP conjugated secondary antibodies and ECL reagents (Millipore), and quantitated with ImageJ software (NIH, version 1.38x).

### Immunofluorescence microscopy

Cells were fixed with 3.7% paraformaldehyde in PBS, permeabilized with acetone in PBS, blocked with BSA, incubated with anti-Robo1 antibody (Abcam), stained with a FITC conjugated goat anti-rabbit antibody (Invitrogen, Alexa 488, A-11008), rinsed in PBS, and then immersed in PBS containing 1ug/ml Hoechst 33258 for 1 min. Images were obtained with a Carl Zeiss LSM510 meta confocal microscope equipped with a Plan-Apochromat 63X objective. Argon (ex:488nm), and UV (ex:364nm) lasers were used with appropriate band pass filters (BP:505-530, BP:420-480) to detect Alexa 488 and Hoechst 33342 respectively.

### Cdc42/Rac activity assays

Cdc42 activation was measured by affinity precipitation of cellular GTP-bound form of Cdc42 as described [20]. Cells were untreated or treated with 60nM IgG or R5 antibody for 8 hours. Cells were lysed (50 mM Tris, pH 7.4, 1% Triton X-100, 150 mM NaCl, 10 mM MgCl_2_, 0.5% sodium deoxycholate, 0.1% sodium dodecyl sulfate (SDS), 10% glycerol, 10 μg/ml each of leupeptin and aprotinin, and 0.1 mM PMSF) and incubated with GST fused to the Cdc42/Rac (p- 21)-binding domain of PAK bound to glutathionecoupled agarose beads (GST-PBD) for 90 minutes at 4°C. The fusion protein beads with bound proteins were then washed three times in lysis buffer, eluted in sample buffer and analyzed by western blotting.

### Cell growth and migration assays

Cells (10,000 per well) were plated in standard tissue culture treated 12-well plates (Falcon, 3043) or in ultra low attachment 24-well plates (Corning, 3473) to assay anchored and nonanchored growth, respectively, as previously described [15,16,18]. Cell numbers were determined by Coulter counter at the indicated time points. Cell migration was measured by a wound healing assay in which confluent layers were scratched with a plastic pipette tip, washed with fresh medium, and cultured for 24 hours. The number of cells entering the wound were counted from phasecontrast images obtained with a digital camera (AxioCam MRm) attached to a Zeiss microscope (Axiovert 40 CFL) equipped with Axiovision 4.5 software as described [21].

## SUPPLEMENTAL FIGURES

Supplementary Figure S1

Supplementary Figure S2

Supplementary Figure S3
