# Supplementary figures and images for: Src activates Abl to augment Robo1 expression in order to promote tumor cell migration
Source: Oncotarget. 2010 Jul 20;1(3):198–209. doi: 10.18632/oncotarget.126 (PMC3058788; doi:10.18632/oncotarget.126)

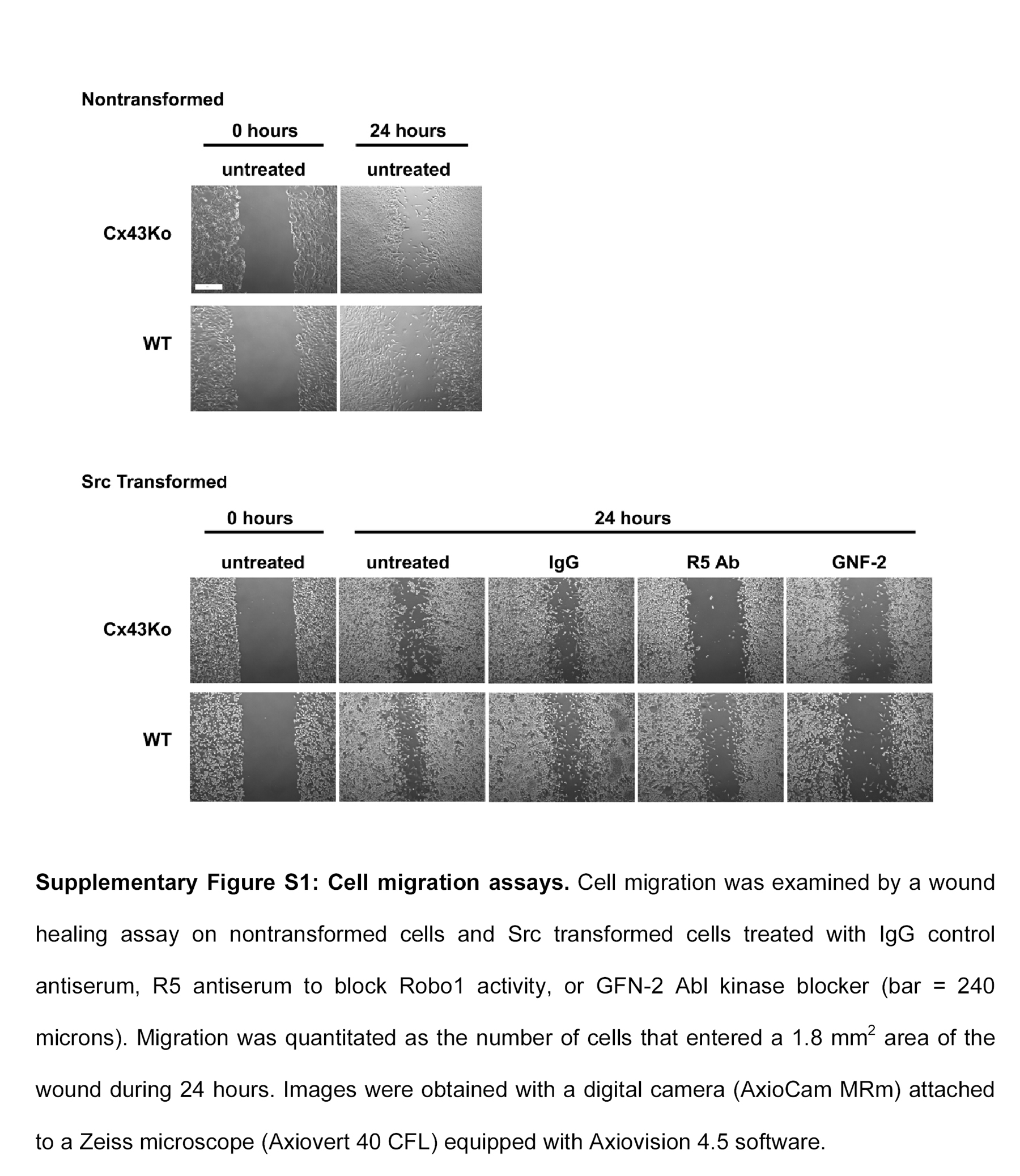

Supplement: Supplementary Figure S1 [file oncotarget-01-198-s001.tif]

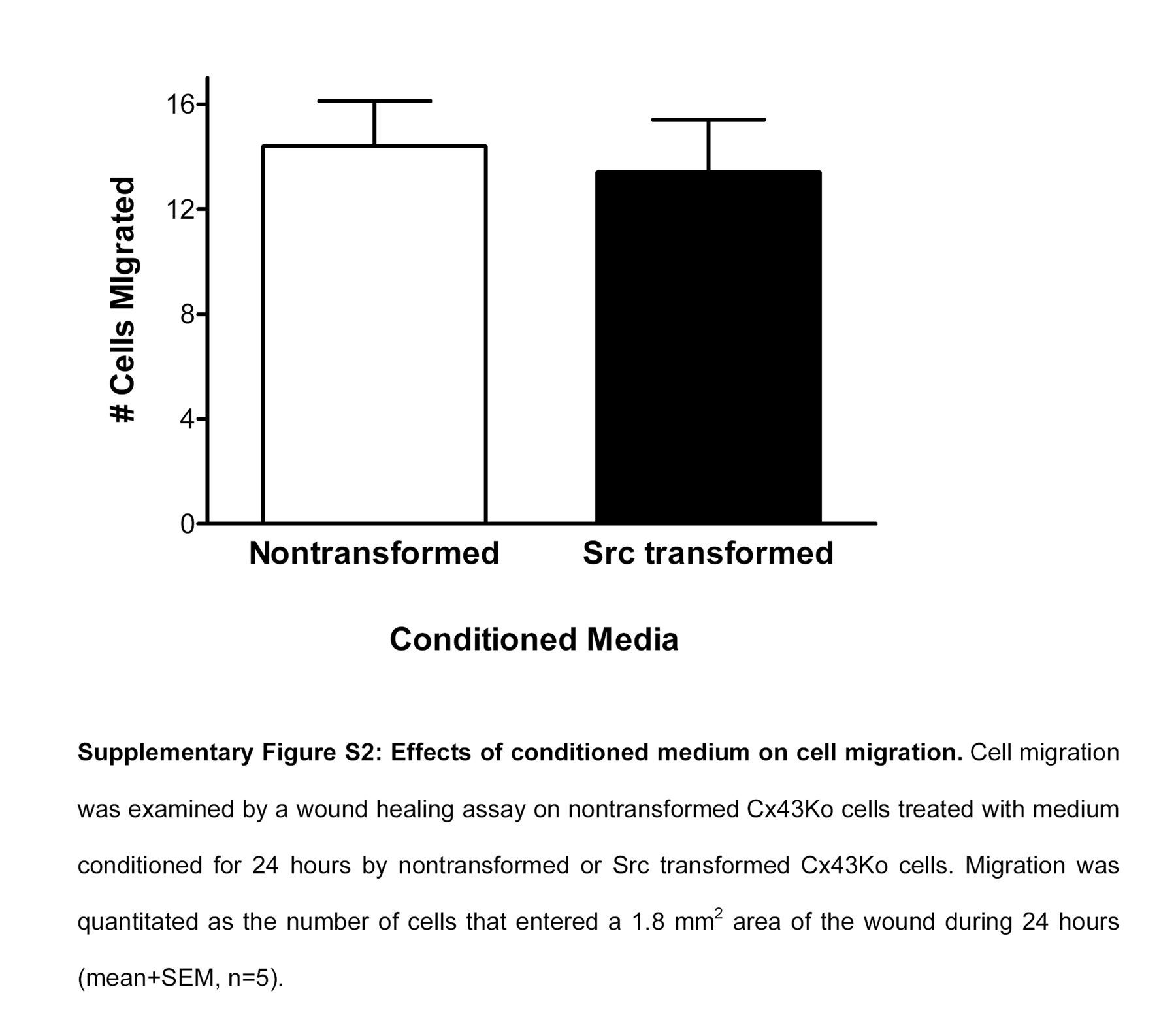

Supplement: Supplementary Figure S2 [file oncotarget-01-198-s002.tif]

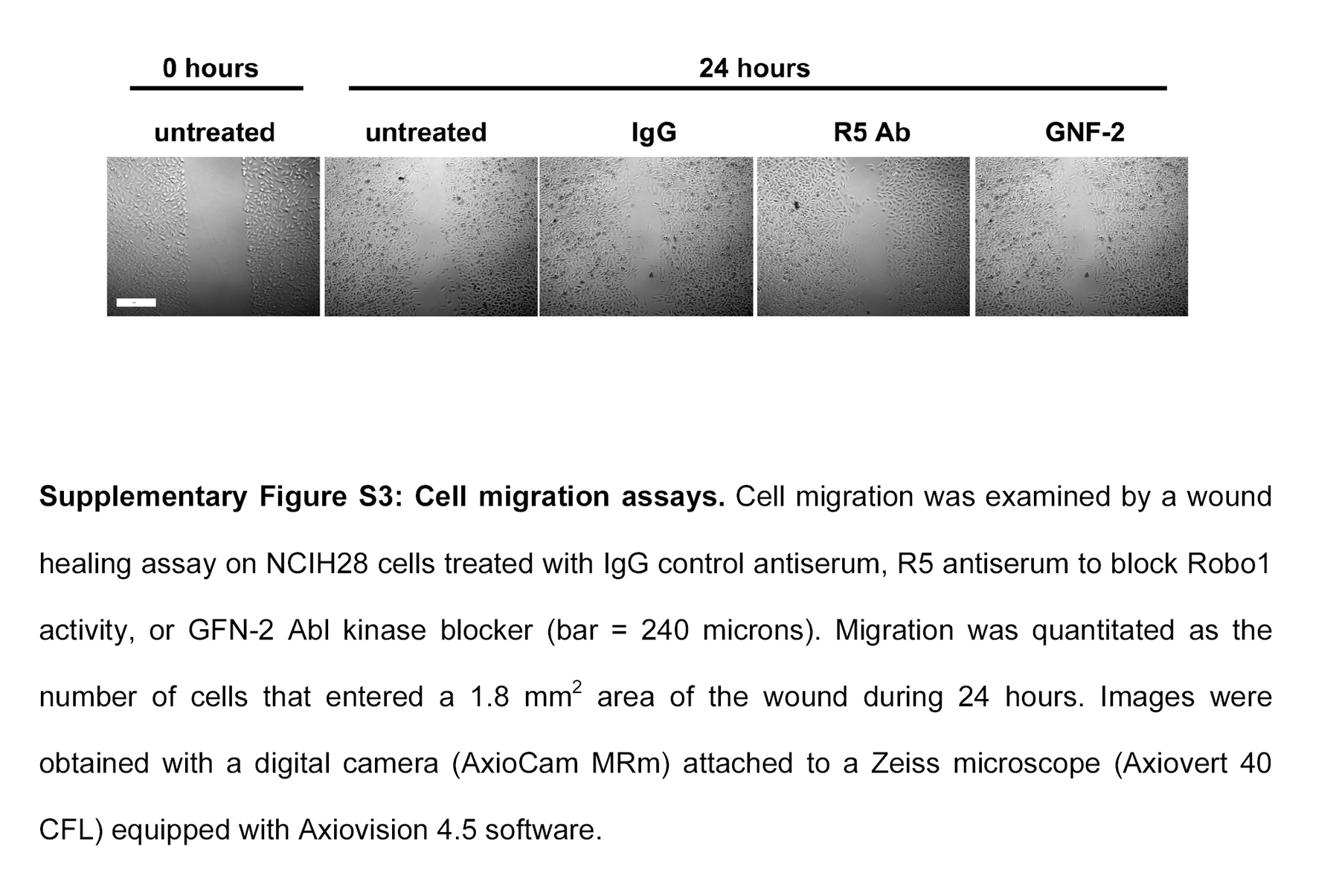

Supplement: Supplementary Figure S3 [file oncotarget-01-198-s003.tif]
